# Adjuvant trastuzumab: progress, controversies, and the steps ahead

**DOI:** 10.3747/co.v13i1.91

**Published:** 2006-02

**Authors:** A.A. Joy, J.R. Mackey

**Affiliations:** * Department of Oncology, University of Alberta, and the Cross Cancer Institute, Edmonton, Alberta

**Keywords:** Early-stage breast cancer, trastuzumab, adjuvant therapy, docetaxel, paclitaxel, carboplatin, doxorubicin

## Abstract

*The* major breast cancer story of 2005 was trastuzumab, a monoclonal antibody directed against the Her-2 oncoprotein, and how it greatly improves outcomes for women with *HER2-*positive early-stage breast cancer. With early results showing that use of the drug can prevent roughly one half of relapses, adjuvant trastuzumab has been approved, funded, and accepted as the standard of care in many Canadian jurisdictions. In the present brief report, we summarize the four major adjuvant trials, outline some key controversies, and suggest steps to provide more-effective and better-tolerated adjuvant systemic therapy for the relevant patient subgroup.

## 1. INTRODUCTION

Improvements in breast cancer detection and treatment have contributed to declining breast cancer–specific mortality rates since the mid-1990s 1,2. Still, outcomes of early-stage breast cancer remain suboptimal: women experience relapses despite state-of-the-art adjuvant chemotherapy and hormonal therapy, and distant metastatic disease most commonly leads to premature death.

In 2005, we witnessed a revolution in adjuvant breast cancer therapy, with reports of major reductions in risk of recurrence with the use of a biologically targeted agent. In the present brief report, we summarize the early results from four large-scale adjuvant trastuzumab studies, attempt to place the currently available treatment options in context, and discuss how controversies will be addressed in upcoming clinical trials. Ultimately, we hope that these discussions will assist Canadian health care providers and their breast cancer patients in making the treatment decisions that they now face on a daily basis.

## 2. DISCUSSION

### 2.1 The Her-2 Pathway

A growing understanding of breast cancer biology has identified new targets for therapy. We now appreciate that growth factor receptors can trigger intra-cellular signalling cascades that modulate the cell cycle and apoptosis, influencing the behaviour of breast cancer cells. Amplification of the human epidermal growth factor receptor–2 gene (*HER2*) leads to an overabundance of the Her-2 protein on the cell surface, enhances dimerization of Her-2 with itself or with other members of the epidermal growth factor receptor (egfr) family, and triggers intracellular tyrosine kinase activity and signal transduction pathways. The ultimate result of this Her-2 driven signalling is inhibition of apoptosis and promotion of cell growth, division, angiogenesis, and metastasis.

The *HER2* alteration occurs in 20% – 25% of human breast tumours 3,4 and can be identified by protein overexpression as assessed using immunohistochemistry (ihc) or by gene amplification as assessed using fluorescence in-situ hybridization (fish) or chromogenic in-situ hybridization. Disease that is *HER2-*positive is associated with negative clinical prognostic factors such as high tumour grade, dna aneuploidy, a high cell proliferation rate, estrogen or progesterone negativity (or both), and *TP53* gene mutation. Aberrations in a variety of other molecular biomarkers of breast cancer invasiveness and metastasis have also been seen 5–7. As a result, breast cancers with *HER2* overexpression have a more aggressive disease course and poorer overall prognosis than are seen with *HER2-*negative breast cancers.

### 2.2 Trastuzumab

Trastuzumab (Herceptin: Genentech, San Francisco, CA, U.S.A.) is a humanized monoclonal antibody directed against the extracellular portion of the Her-2 protein. Trastuzumab acts by activating antibody-dependent cellular cytotoxicity, directly disrupting receptor dimerization and downstream effector signalling, and internalizing and degrading the receptor. In model systems, trastuzumab reduced cellular proliferation, suppressed angiogenesis, and ultimately caused cell cycle arrest 8.

Patients with advanced *HER2-*positive cancers benefit from trastuzumab therapy delivered either as a single agent or in combination with chemotherapy. In metastatic breast cancer, an objective response to first-line single-agent trastuzumab was seen in 26% of patients 9; however, in patients who had received prior chemotherapy, the response rate was substantially lower (approximately 12% – 15%) 10,11. Outcomes—including response rate, time to progression, and overall survival—are improved when trastuzumab is given concurrently with chemotherapy 12–14.

### 2.3 The Major Adjuvant Trastuzumab Trials

#### 2.3.1 Study Designs and Efficacy Signals

**Figure 1:**
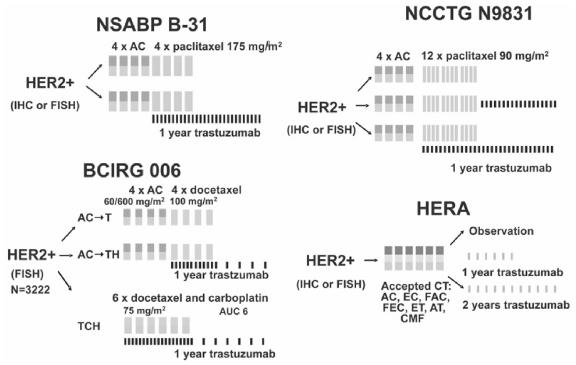
Adjuvant trastuzumab study designs. ac = doxorubicin–cyclophosphamide; t = docetaxel; h = trastuzumab; at = doxorubicin–taxane; auc = area under the curve; bcirg = Breast Cancer International Research Group; cmf = cyclophosphamide–methotrexate–5-fluorouracil; ct = chemotherapy; ec = epirubicin-cyclophosphamide; et = epirubicintaxane; fac = fluorouracil–doxorubicin-cyclophosphamide; fec = fluorouracil–epirubicin–cyclophosphamide; fish = fluorescence in-situ hybridization; ich = immunohistochemistry; ncctg = North Central Cancer Treatment Group; nsabp = National Surgical Adjuvant Breast and Bowel Project; hera = Herceptin Adjuvant Trial.

Four large-scale, randomized phase iii studies are evaluating the role of trastuzumab in the adjuvant setting. Figure 1 depicts the design of those studies. Descriptions of the trial populations, efficacy signals, and cardiac safety data have been reported for each study (Tables I – III).

##### National Surgical Adjuvant Breast and Bowel Project (nsabp) B31 Trial and North Central Cancer Treatment Group (ncctg) N9831 Trial

The nsabp B31 protocol (Figure 1) randomized 1736 axillary node–positive, *HER2-*positive breast cancer patients to 4 cycles of standard doxorubicin and cyclophosphamide (ac) followed by 4 cycles of paclitaxel administered every 3 weeks (ac–p), or the same chemotherapy with weekly trastuzumab starting concurrently with the paclitaxel (ac–ph). In a somewhat similar approach, ncctg N9831, a three-armed study, randomized 1615 patients to 4 cycles of ac chemotherapy followed by weekly paclitaxel (×12 weeks) with or without weekly trastuzumab (×1 year) and allowed for comparison of concurrent (that is, *with* paclitaxel) versus sequential (that is, *after* completion of all chemotherapy) trastuzumab therapy. The populations in the two studies varied somewhat, with N9831 including patients with high-risk node-negative disease.

Although accrual had not finished, similarities in the designs of these two studies prompted the investigators to perform a non-protocol (but U.S. Food and Drug Administration–approved) combined interim analysis 15. With a combined median follow-up of 2.0 years (2.4 years in nsabp B31 and 1.5 years in ncctg N9831), significant benefits were observed in the patients receiving adjuvant trastuzumab. The primary endpoint of disease-free survival (dfs) was improved in the trastuzumab-containing arms [hazard ratio (hr): 0.48; *p* = 3×10^−12^) of the trials, and remained so in all subsets. Preliminary data also suggested longer overall survival in trastuzumab-treated patients (hr: 0.67; 2*p* = 0.015).

##### Breast Cancer International Research Group (bcirg) 006 Trial

For combination with trastuzumab, the bcirg 006 trial selected docetaxel-based chemotherapy. This choice was made because of docetaxel’s clinical efficacy in advanced breast cancer and the suggestion of preclinical synergy with docetaxel, carboplatin, and trastuzumab 13,16,17. The protocol randomized 3222 women with *HER2-*amplified, node-positive or high-risk node-negative operable breast cancer to one of three study arms (Figure 1):

- Arm 1: 4 cycles of standard ac chemotherapy followed by 4 cycles of docetaxel administered every 3 weeks (ac–t)
- Arm 2: 4 cycles of standard ac chemotherapy, followed by 4 cycles of docetaxel with concurrent weekly trastuzumab; then trastuzumab every 3 weeks to complete 1 year of antibody therapy (ac–th)
- Arm 3: Docetaxel–carboplatin–trastuzumab for 6 cycles, followed by trastuzumab every 3 weeks to complete 1 year of antibody administration (tch)

All patients had *HER2-*amplified disease (fish+) as assessed by a centralized laboratory (Table II). The study population also included high-risk node-negative disease (even if the primary tumours were small).

The occurrence of 300 dfs events triggered a protocol-specified interim analysis of bcirg 006. With a median follow-up of 23 months, adding trastuzumab to docetaxel-based chemotherapy improved the primary endpoint of dfs. The ac–th regimen produced a 51% reduction in risk of recurrence [95% confidence interval (ci): 35% to 65%; *p* < 0.00001]. The tch regimen produced a 39% reduction in risk of recurrence (95% ci: 21% to 53%; *p* = 0.0002).

The ac–th regimen had a greater numerical benefit, but the two experimental trastuzumab-containing arms were not statistically different in terms of efficacy. Further follow-up will be required to address the relative efficacies of the two trastuzumab-containing regimens 18.

An intriguing protocol-specified molecular correlative study found that, although all tumours had *HER2* amplification, one third also had amplification of the topoisomerase ii&agr; (*TOP2A*) gene. The Top-2A protein is the main molecular target of anthracycline therapy. Patients with co-amplification of *HER2* and *TOP2A* had a very high dfs when treated with ac–th; the patients without *TOP2A* amplification did not seem to benefit from anthracyclines. Those patients had very good outcomes with the non- anthracycline tch regimen 19.

##### Herceptin Adjuvant Trial (hera)

The hera international clinical trial is led by the Breast International Group. More than 5000 *HER2-*positive patients were randomized into one of three arms following completion of adjuvant or neoadjuvant chemotherapy. Patients received either a standard approach (observation with no further treatment) or trastuzumab administered every 3 weeks. The patients receiving trastuzumab were randomly assigned to either 1 or 2 years of therapy. Efficacy results for the standard approach and the 1-year trastuzumab therapy treatment arms have been reported. With a median follow-up of only 1 year, improvement in the primary endpoint of dfs was seen in the patients who received trastuzumab (2-year dfs: 85.8% vs. 77.4%; hr: 0.54; 95% ci: 0.43 to 0.67; *p* < 0.0001) 20.

#### 2.3.2 Cardiotoxicity with Adjuvant Trastuzumab

Trastuzumab is generally well tolerated, and immediate reactions such as infusion-related hypersensitivity are relatively rare. Trastuzumab-associated cardiotoxicity, however, is a major safety concern 21. Most commonly, it presents as an asymptomatic reduction in cardiac ejection fraction and, less frequently, as overt congestive heart failure (chf).

Type ii chemotherapy–related cardiac dysfunction (crcd)—as trastuzumab-associated cardiotoxicity has been recently named 22—does not occur in all individuals exposed to trastuzumab, does not appear to be dose-related, and when present, is variable in its severity. Type ii crcd has no identifiable cardiac ultra-structural abnormalities such as those seen in patients previously exposed to anthracyclines 23. Standard medical management of trastuzumab-associated chf results in significant symptom improvement, with most patients (approximately 80%) achieving improvement in symptoms with treatment 24. It appears that a major risk factor for the development of type ii crcd is prior or concurrent anthracycline exposure.

The four reported adjuvant trastuzumab studies produced substantially different cardiac safety signals (Table III). It is difficult to determine how much of the variability in the rates of grade 3 and 4 chf is attributable to differences in the study populations (Table I) and how much to the varying ways of identifying and coding cardiac events. Nonetheless, cardiac signals are clearly evident.

The highest reported cardiotoxicity rates occurred in the nsabp B31 and ncctg N9831 trials, where ac is followed by concurrent administration of paclitaxel and trastuzumab (3.5% – 4.1% rate of grade 3 and 4 chf) 15,25. Approximately 15% of patients did not complete the planned 1-year duration of trastuzumab because of asymptomatic declines in left ventricular ejection fraction (lvef). Although the “reversibility” of this cardiotoxicity and its long-term outcomes will require more follow-up, the reported experience is that most patients with cardiotoxicity experience resolution of symptoms after discontinuing trastuzumab and starting medical therapy.

Elimination of the anthracyclines in the bcirg 006 study markedly reduced the rates of chf from 1.7% on the ac–th arm to 0.4% on the tch arm. Although average asymptomatic decreases in lvef on ac–th appeared to persist after treatment, tch appeared to allow full recovery of mean lvef to baseline after completion of treatment.

By limiting enrolment to women with a post-chemotherapy lvef of 55% or more, and by giving trastuzumab after completion of chemotherapy, the hera trial produced a very low chf rate of 0.5%.

#### 2.3.3 Selecting an “Optimal” Adjuvant Trastuzumab Approach

Currently, breast cancer patients and their physicians have a choice of several adjuvant trastuzumab approaches. Head-to-head comparisons of these treatment approaches are not available. In particular, the critical comparison of sequential (arm 2) versus concurrent (arm 3) trastuzumab in the N9813 study is not yet sufficiently mature to determine the best approach (although early results suggest superiority for concurrent administration 26). We are thus in the unsatisfying position of making cross-trial comparisons, which are fraught with the problems of varying trial design (Figure 1), varying patient populations, and variability in eligibility based on tumour size, axillary node status, and *HER2* assessment method (Table I).

The benefits for concurrent administration of trastuzumab with paclitaxel, as per the combined analysis, include clear efficacy, a demonstrated survival advantage, early integration of trastuzumab (which might reduce very early relapses), and a relatively short 15-month duration of infusional therapy. Potential downsides include the highest reported risks of grades 3 and 4 chf (between 3.5% and 4.1%) and longer infusion chair times. For methodology purists, concerns include the difficulties of interpreting a trial in which patients are still being accrued at the time of analysis, and the question of whether the trial-mandated crossover of control patients to adjuvant trastuzumab might obscure long-term safety and efficacy signals.

The potential benefits for concurrent administration of trastuzumab with docetaxel include early integration of trastuzumab with potentially synergistic chemotherapy (which, again, might reduce very early relapses), the shortest 12-month duration of infusional therapy with tch, and less infusion chair time. Although not directly comparable, the risk of cardiotoxicity with the docetaxel-based regimens appears to be less than that seen with paclitaxel, and the tch regimen appears to be the least cardiotoxic option among the various regimens (Table III). Should further follow-up confirm the predictive value of *TOP2A* gene amplification, co-amplification of *TOP2A* may be a useful molecular marker to rationally separate patients who require anthracyclines (and thus make the higher cardiotoxicity rates of ac–th somewhat more acceptable) from patients who would be better treated with tch.

The potential benefits of administering trastuzumab after chemotherapy include a lower risk of grades 3 and 4 chf (0.5% in the hera study, where trial entry required a post-chemotherapy lvef of 55% or more, and 2.5% in the second arm of N9831, where trastuzumab was given to women who, after weekly paclitaxel, had an lvef of 50% or more). The duration of total infusional therapy varies with the chosen chemotherapy regimen in hera, ranging from 15 months to 18 months. The hera approach allows considerable flexibility in the selection of chemotherapy regimens, and the trial allowed aggressive anthracycline–taxane and cyclophosphamide–methotrexate–fluorouracil regimens. The possible downsides of this sequential approach include the theoretic risk of early relapse before trastuzumab is begun and the potential loss of synergistic chemotherapy–trastuzumab interactions. Fortunately, some of these issues will be clarified by a more mature comparison of the N9831 study arms 2 and 3.

## 3. FUTURE DIRECTIONS

Adjuvant therapy for *HER2-*driven breast cancer continues to evolve. Although dfs is dramatically improved in all reported studies, more time is needed to definitively address effects on overall survival. Similarly, only time will tell whether cardiotoxicity is a transient and reversible complication of anthracycline and trastuzumab exposure, or whether irreversible cardiac damage will require long-term chf therapy.

The optimal trastuzumab timing (with or after chemotherapy) is unclear, and the optimal duration of trastuzumab is also unknown. The N9831 study should address the former question, but the most appropriate duration of therapy will be answered, in part, by the comparison of the 1-year and 2-year trastuzumab arms in hera. In the meantime, a small study (*n* = 231 *HER2-*amplified patients) performed by Finnish investigators suggested that 9 weeks of trastuzumab given concurrently with 3 cycles of docetaxel reduced risk of recurrence by approximately 50% 27. That study will undoubtedly spur trials of short-duration versus standard 1-year adjuvant trastuzumab. Whether adjuvant trastuzumab would benefit a woman in the absence of chemotherapy is unknown: proposed studies will explore whether postmenopausal women with estrogen receptor–positive, *HER2-*positive breast cancer treated with aromatase inhibitors alone benefit from the addition of trastuzumab.

Most interesting, however, is how prognosis in *HER2-*driven breast cancer might be further improved. Preclinical and correlative studies are attempting to identify mechanisms of trastuzumab resistance. Studies are in design to explore the relative merits of trastuzumab and the dual egfr/Her-2 tyrosine kinase inhibitor, lapatinib, in early breast cancer. However, an increasing body of preclinical and clinical evidence shows that *HER2* amplification drives proangiogenic signals mediated, in part, by vascular endothelial growth factor 28. Thus, to further improve the outlook for patients with *HER2-*driven breast cancers, a strong rationale exists to pursue studies of combination anti-*HER2* and anti-angiogenic therapy.

Trastuzumab represents the first major success of rationally-designed breast cancer therapy, but the story of *HER2-*targeted breast cancer therapy is by no means over. We expect that the best is yet to come.

## Figures and Tables

**Table I:** Overview of the trial populations in early breast cancer trastuzumab studies (stage i–iiia, mo, invasive adenocarcinoma)

	*N0*							
	*T1a*	*T1b*	*T1c*	*N+*	*ihc*	*fish*	*Randomized*	*ef*
nsabp B31				✓	✓	✓	Pre-chemo	≥50%
Ncctg			✓	✓	✓	✓	Pre-chemo	≥50%
bcirg 006	✓	✓	✓	✓		✓	Pre-chemo	≥50%
hera			✓	✓	✓	✓	Post-chemo	≥55%

N0 = node-negative disease; N+ = node-positive disease; ich = immunohistochemistry; fish = fluorescence in-situ hybridization; ef = left ventricular ejection fraction required for study entry; nsabp = National Surgical Adjuvant Breast and Bowel Project; ncctg = North Central Cancer Treatment Group; bcirg = Breast Cancer International Research Group; hera = Herceptin Adjuvant Trial.

**Table II:** Efficacy in early breast cancer trastuzumab studies

		*bcirg006*		
	*nsabpB31,ncctgN9831*^a^	*ac* → *th*	*tch*	*hera*
Regimen	Concurrent	Concurrent		Sequential
Follow-up	2 Years	2 Years		1 Year
Evaluable patients (*n*)	3351	3222		3387
Hazard ratios				
dfs	0.48 2*p=*3×10^−12^	0.49 *p<*0.0001	0.61 *p=*0.0002	0.54 *p<*0.0001
ddfs	0.47 2*p=*8×10^−10^	NR	NR	0.51 *p<*0.0001
os	0.67 2*p=*0.015	NR	NR	0.76 *p=*0.26

a Combined analysis.

nsabp = National Surgical Adjuvant Breast and Bowel Project; ncctg = North Central Cancer Treatment Group; bcirg = Breast Cancer International Research Group; hera = Herceptin Adjuvant Trial; ac → th = doxorubicin–cyclophosphamide/docetaxel–trastuzumab; tch = docetaxel–carboplatin–trastuzumab; dfs = disease-free survival; ddfs = distant dfs; os = overall survival.

**Table III:** Cardiac safety in early breast cancer trastuzumab studies

	*nsabp B31*^a^		*ncctg N9831*^b^			*hera*		*bcirg 006*^c^		
	*ac → p*	*ac → ph*	*ac → p*	*ac → ph*	*ac → p → h*	*Nil*	*h (1 year)*	*ac → t*	*ac → th*	*tch*
chf [*n* (%)]	4 (0.8)	31 (4.1)	0 (0.3)	20 (3.5)	16 (2.5)	0 (0)	9 (0.5)	3 (0.3)	17 (1.7)	4 (0.4)
Cardiac death (*n*)	1	0	1	1	1	1	0	0	0	0

a Cumulative rates 24.

b Cumulative rates 25.

c Raw rates.

nsabp = National Surgical Adjuvant Breast and Bowel Project; ncctg = North Central Cancer Treatment Group; hera = Herceptin Adjuvant Trial; bcirg = Breast Cancer International Research Group; ac = doxorubicin–cyclophosphamide; p = paclitaxel; h = trastuzumab; t = docetaxel; tch = docetaxel–carboplatin–trastuzumab; chf = New York Heart Association class 3 and 4 congestive heart failure.
